# Effects of tillage, crop establishment and diversification on soil organic carbon, aggregation, aggregate associated carbon and productivity in cereal systems of semi-arid Northwest India

**DOI:** 10.1016/j.still.2019.03.005

**Published:** 2019-07

**Authors:** H.S. Jat, Ashim Datta, M. Choudhary, A.K. Yadav, V. Choudhary, P.C. Sharma, M.K. Gathala, M.L. Jat, A. McDonald

**Affiliations:** a ICAR-Central Soil Salinity Research Institute (ICACSSRI), Karnal, Haryana, India; b Sri Karan Narendra Agriculture University, Jobner, Rajasthan, 303329, India; c International Maize and Wheat Improvement Centre (CIMMYT), Dhaka, Bangladesh; d International Maize and Wheat Improvement Centre (CIMMYT), NASC Complex, Pusa, New Delhi, India; e International Maize and Wheat Improvement Centre (CIMMYT), Kathmandu, Nepal

**Keywords:** ZT, zero tillage, CT, conventional tillage, CA, conservation agriculture, Sc, scenario, AAC, aggregate associated carbon, WSMa, water stable macroaggregates, TWSa, total water stable aggregates, MAC, macroaggregate associated carbon, MicC, microaggregate associated carbon, Soil organic carbon, Aggregate associated carbon, Particulate organic carbon, Crop productivity, Crop management

## Abstract

Intensive tillage based management practices are threatening soil quality and systems sustainability in the rice-wheat belt of Northwest India. Furthermore, it is accentuated with puddling of soil, which disrupts soil aggregates. Conservation agriculture (CA) practices involving zero tillage, crop residue management and suitable crop rotation can serve as better alternative to conventional agriculture for maintaining soil quality. Soil organic carbon is an important determinant of soil quality, playing critical role in food production, mitigation and adaptation to climate change as well as performs many ecosystem functions. To understand the turnover of soil carbon in different forms (Total organic carbon-TOC; aggregate associated carbon-AAC; particulate organic carbon- POC), soil aggregation and crop productivity with different management practices, one conventional agriculture based scenario and three CA based crop management scenarios namely conventional rice-wheat system (Sc1), partial CA based rice-wheat-mungbean system (Sc2), full CA-based rice-wheat-mungbean system (Sc3) and maize-wheat-mungbean system (Sc4) were evaluated. TOC was increased by 71%, 68% and 25% after 4 years of the experiment and 75%, 80% and 38% after 6 years of the experiment in Sc4, Sc3 and Sc2, respectively, over Sc1 at 0–15 cm soil depth. After 4 years of the experiment, 38.5% and 5.0% and after 6 years 50.8% and 24.4% improvement in total water stable aggregates at 0–15 and 15–30 cm soil depth, respectively was observed in CA-based scenarios over Sc1. Higher aggregate indices were associated with Sc3 at 0–15 cm soil depth than others. Among the size classes of aggregates, highest aggregate associated C (8.94 g kg^−1^) was retained in the 1-0.5 mm size class under CA-based scenarios. After 6 years, higher POC was associated with Sc4 (116%). CA-based rice/maize system (Sc3 and Sc4) showed higher productivity than Sc1. Therefore, CA could be a potential management practice in rice-wheat cropping system of Northwest India to improve the soil carbon pools through maintaining soil aggregation and productivity.

## Highlights

• Enrichment in soil organic carbon was observed under ZT diversified cropping system.
• Conservation agriculture improved aggregate indices.
• ZT and crop diversification improved particulate and aggregate associated carbon.
• Conservation agriculture showed higher system productivity.

## 1 Introduction

Rice and wheat are the major cereal crops in the Indo-Gangetic Plains (IGP), grown in rotation on almost 13.5 M ha of land and provides food for 400 million people and a plateauing in its productivity starts appearing since last decade in this region because of fatigued natural resource base (Sharma and Bhushan, 2001). In intensive cropping systems, soil health degradation is a continuous phenomenon in both rainfed and irrigated ecosystems. In rice-wheat cropping system of Northwest India, natural resource degradation coupled with intensive tillage and residue burning is deteriorating the soil quality resulting in low organic carbon, low soil biota, stagnant yields, low factor productivity and farm income. The major forces behind the degradation are both natural and manmade cultivation practices. In human induced soil degradation; tillage, residue removal/burning, faulty irrigation practices and imbalanced application of nutrients played a crucial role (Lal, 2006). In rice-wheat cropping system of South Asia, farmers prefer clean cultivation and tilled the soil more than 15 times in a year which accelerated the soil erosion by breaking down the soil aggregates. Soil structure, the arrangement of soil particles into aggregates and associated pore networks, is the central aspect of soils sustainability in any agro-ecosystems and is an essential feature of soil that allows it to provide many important functions for example acts as a medium for plant growth, a sink of carbon sequestration, and contributes to human health (Lehmann et al., 2017). Soil aggregation is an important mechanism which contributes to soil fertility through reducing soil erosion, mediating air permeability, infiltration and nutrient cycling (Zhang et al., 2012). Soil aggregates play an important role in retention of soil organic carbon and protection against decomposition from various decomposing agents thereby help in long term stabilization of soil organic carbon (SOC) by physical encapsulation (Tisdall and Oades, 1982; Golchin et al., 1994; Six et al., 2000a). The location of SOC in the aggregates and its chemical characteristics also affect the rate of its decomposition (Besnard et al., 1996; Christensen, 1996) and the consequent greenhouse gas (GHG) emissions (Mangalassery et al., 2013). The rate of decomposition of aggregate associated carbon differs among the micro and macroaggregates. Mineralization of C and N can be enhanced by transformation of macroaggregates to the size of microaggregates (Aoyama et al., 1999; Elliott, 1986). Thus the soil organic matter (SOM) associated with the macroaggregates was more labile and less processed than that associated with the microaggregates.

In conventional tillage (CT), soil is tilled several times and the aggregate formation process is disturbed each time due to destruction of aggregates (Six et al., 2002a). During multiple tillage operations, SOM is redistributed within the soil profile and minor changes in it may affect the formation and stability of soil aggregates. A positive linear correlation was reported between SOC content and aggregate size (Carter, 1992), and thus larger aggregates with higher SOC proved effective in minimizing the intensity of slaking and disintegration of aggregates when exposed to water (Blevins et al., 1998). Particulate organic matter (POM) accumulates at soil surface upon crop residues retention under CA and when POM starts decomposing, formation of aggregates in soil occurs (Torres-Sallan et al., 2017).

Besides performing many ecosystem services, SOC is an important parameter which helps in maintaining soil health, sustaining food production, at the same time plays significant role in mitigation, and adaptation to climate change (Francaviglia et al., 2019). Recently there is a growing momentum for action on soil organic carbon in political, financial and technical circles for addressing multiple sustainability goals (Vermeulen et al., 2019). Oldfield et al. (2019) conducted a global meta-analysis and reported positive relationship between soil organic carbon and wheat and maize yield. Conservation agriculture proved to be an excellent alternative to conventional agriculture in the long term of sustainable crop production and SOC sequestration (Gupta Choudhury et al., 2014). In CA, zero-tillage reduces the process of oxidation and loss of soil carbon and nutrients. Crop residues act as barrier between soil and the open environment which may have great role in soil erosion reduction, and soil quality improvement. Soil physical (Indoria et al., 2017), chemical (Jat et al., 2018) and biological (Choudhary et al., 2018a) properties particularly those related to soil carbon sequestration (Xue et al., 2018) are influenced by tillage practices. Dry and wet aggregate size distribution was also improved in zero-tillage (ZT) and residue retention compared to conventional tillage (Govaerts et al., 2009; Lichter et al., 2008). Soil aggregation is affected by quantity and quality of SOC fractions (Lal, 2000) thereby protecting the carbon physically from degradation by enhancing its turn over time in soil (Bajracharya et al., 1997).

Intensive tillage coupled with residue burning/ removal in rice-wheat cropping accentuates soil health deterioration, environmental pollution and soil erosion (Montgomery, 2007) and hampers essential ecosystem functions (Gupta Choudhury et al., 2014) in Northwest India. Therefore, improved CA based management practices would be necessary for enhancing soil quality (Jat et al., 2018) and resilience of the production system to climate change (Gathala et al., 2013). To our knowledge there are no such studies which included the effect of a wide range of CA based indicators on soil aggregation and aggregate associated carbon in Northwest IGP. Therefore, we included a wide range of technologies/ management practices called scenarios (group of technologies/practices) ranging from conventional farmers practice to partial CA and full CA with diversified cropping system adapted to real ground situation of Northwest Indian farmers. We hypothesized that CA based management practices (ZT, residue management with diversified cropping system) will have significant impact on soil aggregation; associated C, POC and systems productivity in cereal(rice/maize)based systems. The specific objectives of this study were (a) to analyze changes in WB-C, TOC, aggregate associated carbon and POC concentrations under different CA based practices over a medium term period (after 4 and 6yrs); (b) to explore the relationship between residue load, TOC, WB-C, aggregate indices and aggregate associated carbon; and (c) to assess the crop productivity with different management practices in Northwest India.

## 2 Materials and methods

### 2.1 Experimental site

This study was conducted at ICAR-Central Soil Salinity Research Institute (ICAR-CSSRI), Karnal, Haryana, India (29°70′N, 76°96′ E). It is situated in the semi-arid region, with average annual rainfall of 700 mm (75–80% of which is received during June–September), daily minimum temperature of 0–4 °C in January, daily maximum temperature of 41–44 °C in June, and relative humidity of 50–90% throughout the year. The experimental site is a reclaimed alkali loam soil and its initial soil characteristics can be obtained from Gathala et al. (2013). The soil was fine-loamy mixed hyperthermic family of *Typic Natrustalf* (Soil Survey Division Staff, 1993).

The site was under a continuous rice-wheat system for ten years before the establishment of the experiment. In May 2009, before the start of the experiment, the entire area was levelled by laser assisted land leveller and uniform puddle transplanted rice was grown in all plots during July–October 2009 to check for and promote site uniformity. The treatments were imposed in rice, maize, wheat and mungbean crops from 2009–2010.

### 2.2 Experimental details

**Fig. 1:**
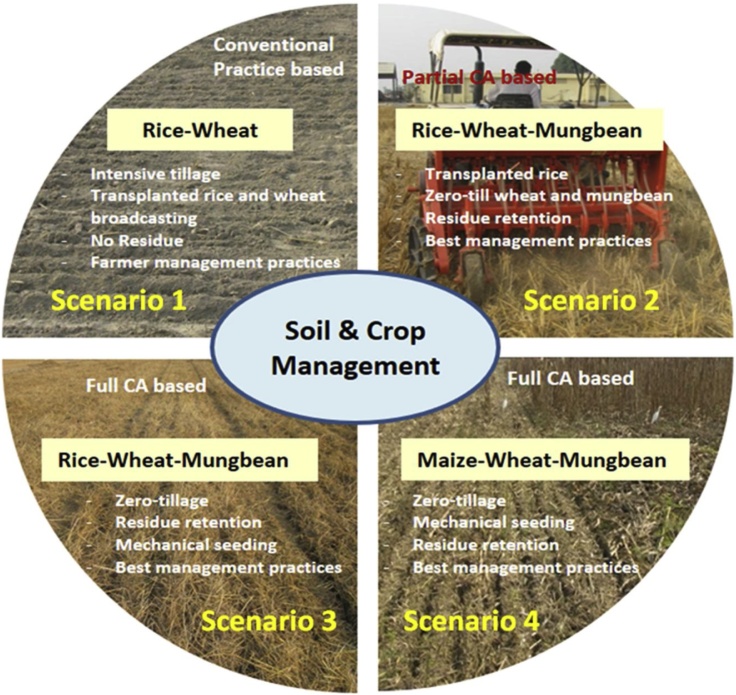
Conventional farmers practice and different CA based agricultural scenarios with soil and crop management practices.

The experiment comprised of four scenarios varying in cropping system, tillage, crop establishment and residue management practices (Fig. 1). The experiment was conducted in a randomized block design and each treatment has three replicates. Each plot size was 100 m × 20 m. The distance between plot to plot was ˜150 cm (buffer zone). In scenario 1 (Sc1-conventional till rice-wheat cropping system, residues removed; business-as-usual), both rice (*Oryza sativa* L.) and wheat (*Triticum aestivum* L.) were sown with conventional tillage, manual transplanting of 30-days-old rice seedlings in puddled soil and wheat by manual broadcasting. Scenario 2 (Sc2- partial CA based rice-wheat-mungbean system) consisted of transplanting of rice in puddled soil and subsequent wheat and mungbean (*Vigna radiata* L.) both by drill seeding in zero-till conditions, residues were incorporated during rice. Under scenario 3 (Sc3- full CA based rice-wheat-mungbean system), crop residues were retained on soil surface and crops (rice, wheat and mungbean) were sown under ZT condition and scenario 4 (Sc4- CA based maize-wheat-mungbean system), all the three crops (Maize: *Zea mays* L., wheat and mungbean) were drill seeded under ZT with residue retention. In all the scenarios best crop management practices for nutrient, water, weed, pests etc. were followed except conventional till rice, where farmer’s practices were followed. In rice, transplanted rice plots were irrigated (5 ± 2-cm depth of standing water) daily for the first one month, and thereafter irrigation (5 to 7-cm depth of standing water) was applied at the appearance of hairline cracks under farmer’s practice. However, with improved management practices (scenario2), continuous flooding of 5 to 7-cm depth for first 15–20 days after transplanting followed by irrigation at -40 to −50 kPa matric potential at 15-cm depth till 1 week before flowering followed by irrigation at -15 to −20 kPa was given in TPR. In zero-tillage direct seeded rice, soil was kept wet for first 20 days followed by irrigation at -20 to −30 kPa matric potential. In maize, wheat and mungbean need based irrigation was applied by targeting the critical crop growth stages.

The nutrients was supplied mainly through urea (46% N), di-ammonium phophate (DAP: 18% N and 46% P), muriate of potash (MoP: 60% K) and NPK complex fertilizer (12:32:16% NPK). In Sc1, 175 + 58 + 0 and 150 + 58 + 0 kg NPK ha^−1^was applied in rice and wheat crop, respectively. In Sc2, Sc3 and Sc4, a fertilizer dose of 151 + 64 + 32 kg NPK ha^-1^ was applied in wheat crop. Whereas in rice, a fertilizer dose (kg NPK ha^−1^) of 151 + 58 + 60 in Sc2, 162 + 64 + 62 in Sc3 was applied. Maize crop was fertilized with a dose of 174 + 64 + 62 NPK ha^−1^ in Sc4. No fertilizer was given in mungbean crop. The complete information on nutrient management can be obtained from Gathala et al. (2013) for all the scenarios.

### 2.3 Soil sampling and analysis

Soil samples were collected from 0 to 15 and 15–30 cm soil depth after 4 and 6 years of the experiment in October, 2013 and 2015 (after rice harvest) from four places in each plot. Then the soil samples were mixed thoroughly to make a composite sample for each plot/replication. The soil samples were air-dried, crushed to pass through a 2-mm sieve, and stored in plastic jars for organic carbon analysis. Oxidizable organic C was determined by Walkley and Black method (Walkley and Black, 1934) termed as Walkley and Black C (WB-C) and total organic carbon (TOC) was estimated by Elementer TOC analyzer (Vario TOC Cube).

For aggregate analysis, separately soil samples (0–15 and 15–30 cm) were collected with the help of a spade from 3 to 4 places of each plot and a composite soil sample (about 500 g) was prepared by proper mixing. Then the samples were air dried and bigger clods were crushed by wooden hammer and stored in granular form (>5-8 mm size). Then 100 g soil was taken for wet sieving analysis. Soil samples passed through 5-mm sieve were used for estimation of aggregate size distribution by wet sieving method (Yoder, 1936) for the separation of four aggregate size classes namely coarse macroaggregate (>2.0 mm), mesoaggregate (2.0–0.25 mm), microaggregate (0.25–0.05 mm) and ‘silt + clay’ sized fractions (<0.05 mm). One sample was kept for determination of water stable aggregates in each set of the Yoder apparatus. However, other was used for estimation of primary particles after dispersion with 0.5% (w/v) sodium hexametaphosphate in 1:3 (soil:solution) ratio. Mechanically stirring of the suspension was done for 15 min before the vertical oscillation of the apparatus for 30 min at a frequency of 50 cycle’s min^−1^ with taking care that the samples on the top sieve remain immersed throughout the full stroke. Before starting the oscillation, soil was left for slaking in water for 2 min. Sieves were then taken out and kept 5 min to drain out the water. The water stable aggregates (without dispersion) and the primary particles (with dispersion) of different sizes were collected from the respective sieves separately and weighed after oven drying at 50^0^C for 24 h. After correction of sand content, the following parameters expressing the status of aggregation were determined:

1. Water stable macro and microaggregates: The macroaggregates were determined by adding the aggregates retained over 0.25–2.0 mm sieves, while the microaggregates referred to aggregates retained on 0.05–0.25 mm sieves.

$$\text{W}\text{S}\text{A}\;(\text{\%})=\frac{\left[\left(\text{W}\text{e}\text{i}\text{g}\text{h}\text{t}\;\text{o}\text{f}\;\text{s}\text{o}\text{i}\text{l}+\text{s}\text{a}\text{n}\text{d}\right)\text{*}i-\left(\text{W}\text{e}\text{i}\text{g}\text{h}\text{t}\;\text{o}\text{f}\;\text{s}\text{a}\text{n}\text{d}\right)\text{*}i\right]}{\text{W}\text{e}\text{i}\text{g}\text{h}\text{t}\;\text{o}\text{f}\;\text{s}\text{a}\text{m}\text{p}\text{l}\text{e}}$$

Where, *i* denote the size of the sieve. The percentage of water stable macroaggregates (WSMa) and water stable microaggregates (WSMia) is the summation of soil aggregate size fractions of >0.25 mm and <0.25 mm, respectively. These two were summed up to estimate the total water stable aggregates.

2. Mean weight diameter (MWD) and geometric mean diameter (GMD):

$$\text{M}\text{W}\text{D}\;(\text{m}\text{m})=\;\frac{\sum_{\text{i}=1}^{\text{n}}\text{X}\text{i}\text{W}\text{i}}{\sum_{\text{i}=1}^{\text{n}}\text{W}\text{i}}$$

$$\text{G}\text{M}\text{D}\;\left(\text{m}\text{m}\right)=\text{e}\text{x}\text{p}\left[\frac{\sum_{i=1}^{n}\text{W}i\;\text{l}\text{o}\text{g}\;\text{X}i}{\sum_{i=1}^{n}\text{W}i}\right]$$

*Where*, n is the number of fractions (0.1-0.25, 0.25-0.5, 0.5–1.0, 1.0–2.0, >2.0 mm), X*i* is the mean diameter (mm) of the sieve size class (0.175, 0.375, 0.75, 1.5 and 2.0 mm) and W*i* is the weight of soil (g) retained on each sieve.

3. Aggregate stability (AS):

$$AS=\frac{\left(\text{P}\text{e}\text{r}\text{c}\text{e}\text{n}\text{t}\;\text{s}\text{o}\text{i}\text{l}\;\text{p}\text{a}\text{r}\text{t}\text{i}\text{c}\text{l}\text{e}\text{s}\;>\;0.25\;\text{m}\text{m}\;-\text{P}\text{e}\text{r}\text{c}\text{e}\text{n}\text{t}\;\text{p}\text{r}\text{i}\text{m}\text{a}\text{r}\text{y}\;\text{p}\text{a}\text{r}\text{t}\text{i}\text{c}\text{l}\text{e}\;>\;0.25\;\text{m}\text{m}\right)}{\text{P}\text{e}\text{r}\text{c}\text{e}\text{n}\text{t}\;\text{p}\text{r}\text{i}\text{m}\text{a}\text{r}\text{y}\;\text{p}\text{a}\text{r}\text{t}\text{i}\text{c}\text{l}\text{e}\;<\;0.25\;\text{m}\text{m}}$$

4. Aggregate ratio (AR):

$$\text{A}\text{g}\text{g}\text{r}\text{e}\text{g}\text{a}\text{t}\text{e}\;\text{r}\text{a}\text{t}\text{i}\text{o}=\;\frac{\text{P}\text{e}\text{r}\text{c}\text{e}\text{n}\text{t}\;\text{o}\text{f}\;\text{w}\text{a}\text{t}\text{e}\text{r}\;\text{s}\text{t}\text{a}\text{b}\text{l}\text{e}\;\text{m}\text{a}\text{c}\text{r}\text{o}\text{a}\text{g}\text{g}\text{r}\text{e}\text{g}\text{a}\text{t}\text{e}\text{s}}{\text{P}\text{e}\text{r}\text{c}\text{e}\text{n}\text{t}\;\text{o}\text{f}\;\text{w}\text{a}\text{t}\text{e}\text{r}\;\text{s}\text{t}\text{a}\text{b}\text{l}\text{e}\;\text{m}\text{i}\text{c}\text{r}\text{o}\text{a}\text{g}\text{g}\text{r}\text{e}\text{g}\text{a}\text{t}\text{e}\text{s}}$$

The total organic C associated with different aggregate size fractions was also determined by using the Elementer TOC analyzer (Vario TOC Cube) and termed as aggregate associated carbon. Particulate organic carbon (POC) was determined by following the method of Cambardella and Elliott (1992). It was determined as a sand-sized fraction (>0.53 μm) of organic matter derived from semi-decomposed above ground crop residues near the soil surface or roots below the soil surface.

### 2.4 Rice equivalent yield

The crops were harvested manually from 4 × 4 m^2^ randomly selected 4 quadrate from each plot and recorded the grain yield. To express the overall impact of scenarios, system productivity was calculated on rice equivalent yield (REY) basis for wheat and mungbean grain yield. Grain yield of crops were recorded at 14% moisture basis. System productivity (t ha^−1^) was computed using below equation.

$$\text{R}\text{E}\text{Y}\;\left(\text{t}/\text{h}\text{a}\right)=\frac{\left\{\text{w}\text{h}\text{e}\text{a}\text{t}/\text{m}\text{a}\text{i}\text{z}\text{e}/\text{m}\text{u}\text{n}\text{g}\text{b}\text{e}\text{a}\text{n}\;\text{y}\text{i}\text{e}\text{l}\text{d}\;(\text{t}/\text{h}\text{a})\;\times \;\text{M}\text{S}\text{P}\;\text{o}\text{f}\;\text{w}\text{h}\text{e}\text{a}\text{t}/\text{m}\text{a}\text{i}\text{z}\text{e}/\text{m}\text{u}\text{n}\text{g}\text{b}\text{e}\text{a}\text{n}\;(\text{I}\text{N}\text{R}\;\text{t}/\text{h}\text{a})\right\}}{\text{M}\text{S}\text{P}\;\text{o}\text{f}\;\text{r}\text{i}\text{c}\text{e}\;(\text{I}\text{N}\text{R}\;\text{t}/\text{h}\text{a})}$$

*Where*, MSP is the Minimum Support Price; INR is the India Rupee

### 2.5 Recycling of crop residues

Scenario 2, Sc3, and Sc4 received a total of 78.5, 74.5 and 96.7 t ha^−1^ crop residues, respectively in six years of study (Table 1). In Sc2, 4.1 to 12.7 t ha^−1^ of anchored rice stubbles and 0.7 to 3.5 t ha^−1^ anchored wheat stubbles were retained over the years at the time of wheat and mungbean sowing, respectively. In rice, 2.5 to 4.7 t ha^−1^ of mungbean residue was incorporated into soil during puddling operation of rice. In Sc3, full rice (3.7 to 10.2 t ha^-1^) and mungbean residues (1.8 to 4.6 t ha^-1^), and anchored wheat stubbles (0.9 to 3.6 t ha^-1^) were retained on the soil surface over the years. In Sc4 similar amounts of crop residues (wheat and mungbean) to Sc3 were retained.

**Table 1:** Residues management (retention/ incorporation) in different scenarios (Mg ha^−1^).

Scenarios	2009-10			2010-11			2011-12			2012-13			2013-14			2014-15			Total
	Rice/maize	Wheat	Mung bean	Rice/maize	Wheat	Mung bean	Rice/maize	Wheat	Mung bean	Rice/maize	Wheat	Mung bean	Rice/maize	Wheat	Mung bean	Rice/maize	Wheat	Mung bean	
Sc1	R	R	F	R	R	F	R	R	F	R	R	F	R	R	F	R	R	F	–
Sc2	4.2	3.5	2.6	4.1	2.6	4.7	7.4	1.6	3.3	10.6	0.7	2.5	10.6	1.8	3.0	12.7	0.9	1.7	78.5
Sc3	6.4	3.6	2.2	10.2	2.8	4.4	7.2	1.6	3	5.3	1.8	4.6	3.7	2.0	3.1	6.9	0.9	1.8	74.5
Sc4	10.0	3.5	2.1	13.7	2.6	3.9	9.5	1.6	2.9	10	1.4	4.6	10.2	1.7	2.8	13.1	1.1	2.1	96.7

Where, R: remove residue; F: fallow; Sc1: conventional rice-wheat system; Sc2: partial CA-based rice-wheat-mungbean system; Sc3: full CA-based rice-wheat-mungbean system; Sc4: full CA-based maize-wheat-mungbean system.

### 2.6 Statistical analysis

The data were subjected to analysis of variance (ANOVA) and using the general linear model (GLM) procedure of the SPSS window version 16.0 (SPSS Inc., Chicago, USA). The figures (graphs) were prepared by using SigmaPlot 11.0 software. Treatment means were separated by Duncan Multiple Range Test at 5% level of significance (*p<*0.05).

## 3 Results

### 3.1 Soil organic carbon

In 2013 (after 4 yrs), Sc4, Sc3 and Sc2 registered 71%, 66.7% and 22% higher WB-C, respectively over Sc1 at 0–15 cm soil depth (Table 2). About 40% higher WB-C (4.9 g kg^−1^) was recorded at 15–30 cm soil depth under Sc2 over other scenarios. Total organic carbon also showed similar trend to WB-C, highest increase was observed in full CA based scenarios (68–71%) over Sc1 at 0–15 cm soil depth. At 15–30 cm soil depth, Sc2 showed highest increment in TOC (about 42%) over Sc1. In 2015 (after 6 yrs), Sc4 recorded 67% and 75% higher WB-C and TOC over Sc1 at 0–15 cm soil depth,respectively. In 2015, at 0–15 cm soil depth, WB-C increased from 6.5% to 23.6% over 2013, highest was associated with Sc2 (23.6%) (Table 2). At lower depth, TOC increased significantly and varied from 29.6% to 47.4% irrespective of scenarios.

**Table 2:** Effect of different CA-based scenarios on oxidizable organic carbon and TOC in different soil layers after 4 (year 2013) and 6 years (year 2015).

	2013				2015			
	WB-C (g kg^−1^)		TOC (g kg^−1^)		WB-C (g kg^−1^)		TOC (g kg^−1^)	
	Soil Layer (cm)							
Scenarios	0-15	15-30	0-15	15-30	0-15	15-30	0-15	15-30
Sc1	4.5^b^	3.5^a^	6.5^b^	3.8^b^	4.9^c^	4.4^b^	6.3^c^	5.6^ab^
Sc2	5.5^b^	4.9^a^	8.1^ab^	5.4^a^	6.8^b^	5.5^a^	8.7^b^	7.0^a^
Sc3	7.5^a^	3.6^a^	10.9^a^	3.8^b^	8.1^a^	4.0^c^	11.4^a^	5.2^b^
Sc4	7.7^a^	3.6^a^	11.1^a^	3.8^b^	8.2^a^	3.4^c^	11.0^a^	5.0^b^

Where, WB-C: walkley and Black Carbon; TOC: total organic carbon; Sc1: conventional rice-wheat system; Sc2: partial CA-based rice-wheat-mungbean system; Sc3: full CA-based rice-wheat-mungbean system; Sc4: full CA-based maize-wheat-mungbean system.

Different small letters within the same column showed the significant difference at *p* <  0.05 according to Duncan Multiple Range Test for separation of mean.

### 3.2 Soil aggregate indices

CA-based partial/full practices (Sc2, Sc3 and Sc4) caused a significant increase of 38.5% and 50.8% in TWSA in upper soil layer (0–15 cm) compared to Sc1 in 2013 and 2015, respectively. At 0–15 cm soil depth under CA based scenarios, the water stable macroaggregates were 67.7% and 86% higher in 2013 and 2015, respectively than Sc1. On average, about 15.6% higher water stable macroaggregates was observed in 2015 after 2 years of continuous CA (Table 3). Mean weight diameter (MWD), GMD, AS and AR also showed similar trend. Sc3 showed higher MWD (2.44 and 2.66 mm), GMD (1.28 and 1.29 mm), AS (41.5 and 45.7%) and AR (4.6 and 7.9) over other scenarios at 0–15 cm soil depth in 2013 and 2015, respectively. On average, CA based scenarios showed 46.8% and 38.5% higher MWD, 66.7% and 84.7% higher AS and 46.5% and 50.2% higher GMD over Sc1 (Table 3) in 2013 and 2015, respectively.

**Table 3:** Effect of different CA-based practices on distribution of different aggregate indices.

		WSMa (%)			WSMia (%)				Total WSA (%)						MWD (mm)					AS (%)								GMD (mm)					AR			
Scenarios		2013		2015	2013	2015			2013			2015			2013		2015			2013			2015					2013		2015			2013		2015	
0-15 cm soil depth																																				
Sc1		21.93^c^		23.46^b^	8.91^a^		9.82^a^		30.84^b^				33.29^b^			1.48^c^			1.67^c^			22.42^d^		24.00^b^		0.76^d^			0.79^c^			0.79^c^			0.89^b^	
Sc2		32.34^b^		42.57^a^	9.10^a^		6.63^b^		41.44^a^				49.20^a^			2.06^b^			2.20^b^			32.61^c^		42.92^a^		0.98^c^			1.15^b^			1.83^bc^			6.00^a^	
Sc3		40.33^a^		44.40^a^	3.44^b^		6.91^b^		43.77^a^				51.32^a^			2.44^a^			2.65^a^			41.54^a^		45.74^a^		1.28^a^			1.29^a^			4.62^a^			7.97^a^	
Sc4		37.65^a^		44.00^a^	5.31^b^		6.10^b^		42.97^a^				50.10^a^			2.02^b^			2.09^b^			37.96^b^		44.36^a^		1.08^b^			1.12^b^			3.05^b^			7.74^a^	
Mean	33.06		38.60		6.61		7.37	39.76			45.98			2.0					2.15			33.63		39.26		1.03			1.09			2.58			5.65	
15-30 cm soil depth																																				
Sc1		17.95^a^		20.28^c^	10.85^a^		8.79^b^			28.80^a^			29.07^c^			1.27^a^		1.12^c^			18.49^a^				20.88^c^		0.62^a^				0.66^a^			0.56^a^		0.69^b^
Sc2		17.54^a^		30.99^a^	12.95^a^		8.43^a^			30.50^a^			39.41^a^			1.31^a^		1.56^a^			17.69^a^				31.24^a^		0.56^a^				0.66^a^			0.55^a^		1.64^a^
Sc3		13.90^b^		24.80^b^	12.49^a^		7.11^a^			26.39^a^			31.91^b^			1.34^a^		1.53^ab^			14.32^b^				25.55^b^		0.49^a^				0.63^a^			0.39^b^		0.99^b^
Sc4		18.89^a^		29.97^a^	14.89^a^		7.22^a^			33.79^a^			37.19^a^			1.33^a^		1.41^b^			19.05^a^				30.22^a^		0.53^a^				0.64^a^			0.62^a^		1.50^a^
Mean		17.07		26.51	12.80		15.39			29.87			34.40			1.31		1.41			17.39				26.97		0.55				0.65			0.53		1.21

Where, WSMa: water stable macroaggregates; WSMia: water stable microaggregates; WSA: water stable aggregates; MWD: mean eight diameter; AS: aggregate stability; GMD: geometric mean diameter; AR: aggregate ratio; Sc1: conventional rice-wheat system; Sc2: partial CA-based rice-wheat-mungbean system; Sc3: full CA-based rice-wheat-mungbean system; Sc4: full CA-based maize-wheat-mungbean system.

Different small letters within the same column showed the significant difference at *p* <  0.05 according to Duncan Multiple Range Test for separation of mean.

### 3.3 Aggregate associated carbon

**Fig. 2:**
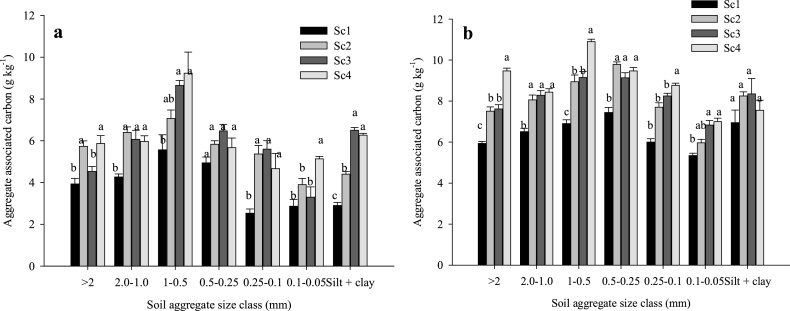
Effects of tillage, cropping system and residue management on soil aggregate associated carbon at 0–15 cm soil depth after rice harvest. a) 2013 (after 4 years) and b) 2015 (after 6 years). Same small letters are not significantly different at P < 0.05 according to Duncan Multiple Range Test for separation of mean. Vertical bars indicate ± S.E. of mean of the observed values. Where, Sc1: conventional rice-wheat system; Sc2: partial CA-based rice-wheat-mungbean system; Sc3: full CA-based rice-wheat-mungbean system; Sc4: full CA-based maize-wheat-mungbean system.

**Fig. 3:**
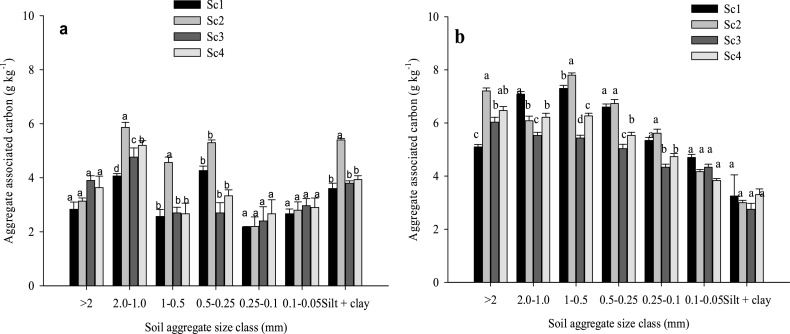
Effects of tillage, cropping system and residue management on soil aggregate associated carbon at 15–30 cm soil depth after rice harvest. a) 2013 and b) 2015. Same small letters are not significantly different at P < 0.05 according to Duncan Multiple Range Test for separation of mean. Vertical bars indicate ± S.E. of mean of the observed values. Where, Sc1: conventional rice-wheat system; Sc2: partial CA-based rice-wheat-mungbean system; Sc3: full CA-based rice-wheat-mungbean system; Sc4: full CA-based maize-wheat-mungbean system.

At 0–15 cm soil depth, the highest (20.4%) and lowest (10.2%) proportion of aggregate associated C was retained with 1.0-0.5 and 0.1-0.05 mm size fractions, respectively in 2013 (Fig. 2a). At 15–30 cm soil depth, 2.0-1.0 mm size particles retained highest proportion (20.1%) of aggregate associated C than others (Fig. 3a). In 2015, highest proportion of aggregate associated C was observed in 1.0-0.5 and 0.5-0.25 mm size class particles (16.3% each) at 0–15 cm soil depth (Fig. 2b) whereas, at 15–30 cm soil depth, 1.0-0.5 mm size class particles retained highest C (17.9%) (Fig. 3b).

**Fig. 4:**
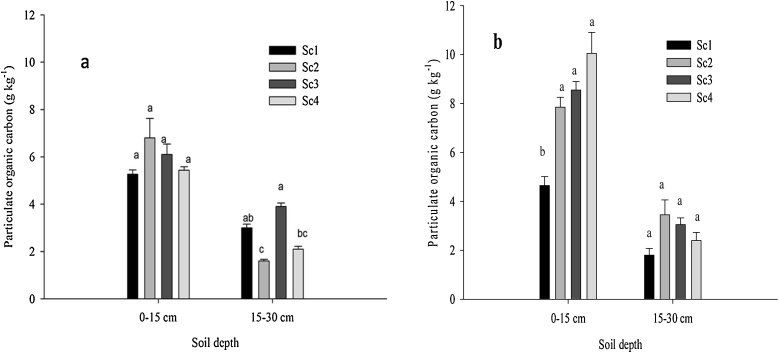
Effects of tillage, cropping system and residue management on particulate organic carbon (POC) after rice harvest. a) 2013 and b) 2015. Same small letters are not significantly different at P < 0.05 according to Duncan Multiple Range Test for separation of mean. Vertical bars indicate ± S.E. of mean of the observed values. Where, Sc1: conventional rice-wheat system; Sc2: partial CA-based rice-wheat-mungbean system; Sc3: full CA-based rice-wheat-mungbean system; Sc4: full CA-based maize-wheat-mungbean system.

**Fig. 5:**
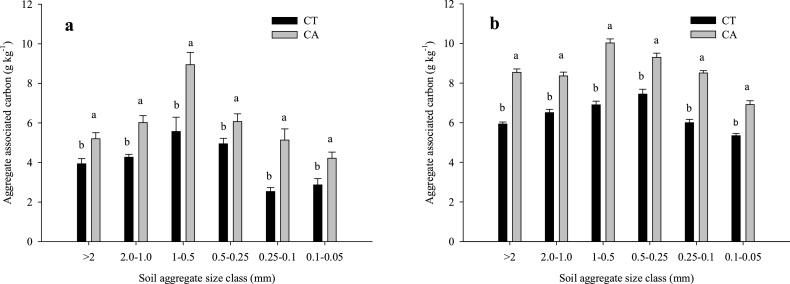
Aggregate associated carbon at 0–15 cm soil depth under conservation agriculture (mean of Sc3 and Sc4) and conventional tillage (Sc1) after rice harvest. a) 2013 and b) 2015. Same small letters are not significantly different at P < 0.05 according to Duncan Multiple Range Test for separation of mean. Vertical bars indicate ± S.E. of mean of the observed values. Where, Sc1: conventional rice-wheat system; Sc2: partial CA-based rice-wheat-mungbean system; Sc3: full CA-based rice-wheat-mungbean system; Sc4: full CA-based maize-wheat-mungbean system.

After 4 years (year 2013) of continuous CA, Sc2 and Sc3 showed 25.9% and 13% higher POC than Sc1 at 0–15 cm soil depth (Fig. 4a). But after 6 years (year 2015), significantly higher POC was observed in CA based scenarios (Sc4- 116%, Sc3-83.8%, Sc2-68.8%) over Sc1 at upper soil layer (Fig. 4b). Silt + clay associated C increased significantly (*p* < 0.05) in all the scenarios in 2015 over 2013 level, highest being associated with Sc1 (140%) at 0–15 cm soil depth (Fig. 2b) although the CA based scenarios registered higher ‘silt + clay’ associated C in both years. Overall, CA-based systems significantly (*p* < 0.05) increased aggregate associated C than CT-based system in both the years (2013 and 2015) (Fig. 5a, b). Among the size classes of aggregates, highest aggregate associated C (8.94 g kg^−1^) was retained in the 1-0.5 mm size class under CA (Fig. 5a). In 2013, under CA based practices highest (103%) aggregate associated C was observed in 0.25-0.1 mm size aggregate class as compared to CT (Fig. 5a). About 89% higher aggregate associated C was observed in 1.0-0.5 mm size aggregate class under CA-based practices in 2015 compared to 2013.

### 3.4 Relations among the residue load and WB-C, TOC, aggregate indices and aggregate associated C

Significant positive correlations among the residue load, WB-C, TOC, aggregate indices and aggregate associated C were observed in both the years (Table 4, Table 5). In 2013, residue load was positively correlated with WB-C (r = 0.81, *p* < 0.05), TOC (r=0.83, *p* < 0.05), WSMa (r=0.89, *p* < 0.05), TWSa (r=0.96, *p* < 0.05), MWD (r=0.78, *p* < 0.05), AS (r=0.87, *p* < 0.05), GMD (r=0.75, *p* < 0.05), AR (r=0.67, *p* < 0.05), POC (r=0.45, *p*<0.05), MAC (r = 0.99, *p* < 0.01), MicC (r=0.94, *p* < 0.05) except water stable microaggregates (Table 4). WB-C and TOC were significantly and positively correlated with all the aggregate indices and aggregate associated C except POC and water stable microaggregates. Water stable aggregates and aggregate indices also showed positive correlations among each other. Similar trend was also observed after 6 years (Table 5).

**Table 4:** Relationships among the SOC, aggregate indices and aggregate associated carbon in 2013.

Pearsons Bivariate Correlations													
	Residue	WB-C	TOC	WSMa	WSMia	TWSa	MWD	AS	GMD	AR	POC	MAC	MicC
Residue	1												
WB-C	0.811*												
TOC	0.826*	0.999**											
WSMa	0.891*	0.941*	0.952*										
WSMia	−0.52	−0.91	−0.9	−0.83									
TWSa	0.962*	0.854*	0.871*	0.967*	−0.66								
MWD	0.779*	0.782*	0.802*	0.938*	−0.75	0.921*							
AS	0.869*	0.939*	0.950*	0.999**	−0.85	0.957*	0.946*						
GMD	0.75*	0.887*	0.898*	0.965*	−0.89	0.893*	0.969*	0.976*					
AR	0.665*	0.899*	0.906*	0.932*	−0.95	0.819*	0.918*	0.947*	0.986*				
POC	0.447*	−0.0	0.034	0.332	0.132	0.508*	0.555	0.326	0.334	0.185			
MAC	0.992**	0.861*	0.875*	0.94*	−0.62	0.986*	0.843	0.924*	0.826	0.752*	0.435		
MicC	0.941*	0.952*	0.961*	0.985*	−0.78	0.970*	0.875	0.977*	0.907*	0.868*	0.285	0.972*	1

Where, WBC: Walkley Black carbon, TOC: Total organic carbon; WSMa: water stable macro aggregates; WSMia: water stable microaggregates; TWSa: total water stable aggregates; MWD: mean eight diameter; AS: aggregate stability; GMD: geometric mean diameter; AR: aggregate ratio; POC: particulate organic carbon; MAC: macroaggregate associated carbon; MicC: microaggregate associated carbon.

* Correlation is significant at the 0.05 level (2-tailed).

** Correlation is significant at the 0.01 level (2-tailed).

**Table 5:** Relationships among the SOC, aggregate indices and aggregate associated carbon in 2015.

Pearsons Bivariate Correlations													
	Residue	WB-C	TOC	WSMa	WSMia	TWSa	MWD	AS	GMD	AR	POC	MAC	MicC
Residue	1												
WB-C	0.925*												
TOC	0.865*	.991**											
WSMa	0.974*	0.938*	0.90*										
WSMia	−0.999**	−0.911	−0.849	−0.976*									
TWSa	0.963*	0.938*	0.905*	0.999**	−0.966*								
MWD	0.673	0.794	0.828*	0.821	−0.677	0.845							
AS	0.964*	0.943*	0.911*	0.999**	−0.966*	1.000**	0.845						
GMD	0.853*	0.894	0.893*	0.949	−0.857	0.961*	0.959*	0.961*					
AR	0.953*	0.986*	0.967*	0.981*	−0.948	0.983*	0.839	0.985*	0.945*				
POC	0.972*	0.964*	0.921*	0.931*	−0.960*	0.92	0.638	0.924*	0.806	0.954*			
MAC	0.772	0.736	0.675	0.628	−0.749	0.601	0.175	0.608	0.395	0.673	0.862*		
MicC	0.957*	0.987*	0.959*	0.936*	−0.943	0.929*	0.702	0.934*	0.843	0.973*	0.994**	0.823*	1

Where, WB-C: walkley black carbon, TOC: total organic carbon; WSMa: water stable macroaggregates; WSMia: water stable microaggregates; TWSa: total water stable aggregates; MWD: mean eight diameter; AS: aggregate stability; GMD: geometric mean diameter; AR: aggregate ratio; POC: particulate organic carbon; MAC: macroaggregate associated carbon; MicC: microaggregate associated carbon.

* Correlation is significant at the 0.05 level (2-tailed).

** Correlation is significant at the 0.01 level (2-tailed).

### 3.5 System productivity

**Fig. 6:**
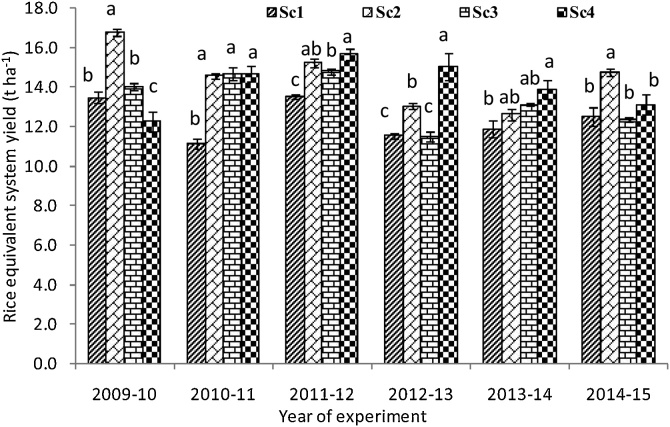
Systems productivity (rice equivalents) over the years as influenced by crop management practices in different scenarios. Same small letters are not significantly different at P < 0.05 according to Duncan Multiple Range Test for separation of mean. Vertical bars indicate ± S.E. of mean of the observed values. Where, Sc1: conventional rice-wheat system; Sc2: partial CA-based rice-wheat-mungbean system; Sc3: full CA-based rice-wheat-mungbean system; Sc4: full CA-based maize-wheat-mungbean system.

The total rice–wheat system grain yield (rice equivalent basis) ranged from 11.12 to 16.76 Mg ha^−1^ across various scenarios and years (Fig. 6). In first year, the highest system productivity was recorded under Sc2 (16.76 t ha^−1^) and Sc3 (13.99 t ha^−1^). Rice based CA systems (Sc2 and Sc3) recorded higher yield over Sc1 during most of the years. Scenario 4 recorded 19.5, 27.6, 22.7, 13.2 and 6.8% higher rice equivalent yield (REY) in the years 2010-11, 2011-12, 2012-13, 2013-14, and 2014-15, respectively over the initial year 2009-10 of experimentation (Fig. 6). On average (6 yrs’ mean), 14.3% and 8.7% higher system productivity was recorded with Sc4 and Sc3, respectively compared to Sc1.

## 4 Discussion

### 4.1 Changes in organic carbon under different CA based practices

Our study showed considerable increase in WB-C, TOC and macroaggregate associated carbon at upper soil layer under CA-based scenarios compared to CT system (Sc1) which may probably due to the recycling of huge quantities of crop residues (Table 1) coupled with ZT further facilitating the stabilization of organic carbon (Six et al., 2002b) as soil organic matter (Lorenz and Lal, 2005; Gupta Choudhury et al., 2014). At 15–30 cm soil depth, higher WB-C and TOC in Sc2 might be due to incorporation of crop residues during puddling in rice which mixes the crop residues in soil every year (Jat et al., 2018). Higher TOC over the years in CA-based scenarios might be due to the decomposition of crop residues with time and leaching of dissolved organic carbon at lower depths through irrigation water (Nachimuthu and Hulugalle, 2016). Higher amount of crop residues coupled with ZT contributed to higher aggregated C concentration in coarse macroaggregates than mesoaggregates and microaggregates. Organic residues in combination with soil particles formed macroaggregates in soil (Tisdall and Oades, 1982). Our findings are in line with Six et al.(2000a, b) and Mikha and Rice (2004) who also reported lower SOC associated with fine particles of <2 mm size class in zero tillage systems. A soil with low organic carbon will be poorly aggregated since the weight and content of organic carbon content in >2.0 mm size class is lower than the weight and content of organic carbon in other classes (Castro Filho et al., 2002). Conservation agriculture with crop residue retention increased TOC, limits soil movement, and thereby enhances soil aggregation (Gupta Choudhury et al., 2014). In CT systems, considerable amount of TOC is lost during the tillage operation due to its disruptive effect and increased soil microbial respiration (Six et al., 2000b).

Higher carbon input in soil through recycling of huge quantities of crop residues coupled with ZT had resulted formation of higher particulate organic carbon (POC) in CA-based scenarios (Fig. 3). In soil, decomposition of particulate organic matter (POM) led to soil aggregation process (Torres-Sallan et al., 2017). During mineralization of POM, bacteria produce mucilage which serves as an adhesive between the mineral particles of the soil and the POM (Tisdall and Oades, 1982). Through the encrustation of POM with mineral particles, SOC becomes enclosed into large macroaggregates (2–8 mm) and small macroaggregates (250–2000 μm) (Torres-Sallan et al., 2017). As POM undergoes further decomposition, it decreases in size and gets encapsulated into microaggregates within macroaggregates (Six et al., 2000b). Higher C associated with‘silt + clay’ sized fraction at 0–15 cm soil depth in both the years under CA based management systems might be due to higher carbon input through residues as well as higher specific surface area of silt and clay serving as sorption sites through cation bridges and ligand exchange (Bandyopadhyay et al., 2010; Gupta Choudhury et al., 2014). Interestingly higher (140%) increment in ‘silt + clay’ associated C was observed in Sc1 in 2015 compared to 2013 level which might be due to the forms of organic carbon which has affinity to bind with ‘silt + clay’ particles upon tillage/puddling or continuous weathering in conventional system. Bandyopadhyay et al. (2010) also observed higher ‘silt + clay’ associated carbon under long term conventional puddling/tillage with green manuring in rice-wheat cropping system. Higher ‘silt + clay’ associated C under long term intensive cultivation with integrated application of fertilizers and organic manure in sorghum-wheat cropping system was also reported by Datta et al. (2018) under Vertisols of semiarid India.

### 4.2 Soil aggregation as influenced by CA based managements

Organic matter is recognized as one of the main precursors in soil particle aggregation. The variation in aggregate size and aggregate indices in tropical soils can be attributed to variation in organic matter content in soil (Castro Filho et al., 2002). CA-based management practices caused a significant increase in total water stable aggregates in surface as well as sub surface soil (Bhattacharyya et al., 2012). Conservation agriculture with residue incorporation or retention combined with either CT or ZT enhanced the formation of water stable aggregates leading to the predominance of macroaggregates compared to microaggregates (Bhattacharyya et al., 2012). Higher water stable macroaggregates in surface soil might be due to the greater accumulation of crop residues at surface soil under CA-based practices (Castro Filho et al., 2002). Microbial biomass and activity was enhanced due to the positive interaction between climatic factors and crop residues in CA-based system (Choudhary et al., 2018a, b) which is directly reflected in soil structure (Oades, 1984). All these facilitated greater stability and size of the aggregates. Higher MWD in CA practices indicates greater number of large aggregates measured in the wet sieving process which might be due to higher accumulation of organic matter (Castro Filho et al., 2002). If the CA-based practices show larger aggregates, then the soil will have higher porosity resulting in higher infiltration which is also reported by Jat et al. (2018) after 4 years of experiment in same scenarios. The GMD directly influences the soil porosity and it represents the dominant aggregate size class of the soil sample (CastroFilho et al., 2002). In CA, besides accumulation of higher organic carbon at surface soil, the absence of soil movement was another important factor contributing to higher GMD (Castro Filho et al., 2002). Therefore, zero tillage coupled with accumulation of organic carbon in upper soil layer through crop residue retention had resulted higher GMD under CA based managements. Cheshire (1979) reported that decomposition of organic matter releases polysaccharides and different organic acids such as humic acids, fulvic acids, humin etc. thereby accentuating stabilization of macroaggregates. Lehmann et al. (2017) provided the evidence of the contribution of soil biota to soil aggregation on macro and microaggregate scales while conducting a global meta-analysis. Choudhary et al. (2018a) also reported higher population of bacteria and fungi which further facilitated the soil aggregation (Lehmann et al., 2017) in CA-based maize-wheat cropping system after 3 years of continuous cultivation in Northwest India. Higher soil aggregation in maize based system might be due to the gel like root exudates of maize released to the soil creating a more stable soil structure around the roots (Naveed et al., 2017). Gupta Choudhury et al. (2014) reported that tillage management had a prominent impact on soil aggregation than residue management and ZT with or without residue showed 46.5% higher water stable macroaggregates in surface soil compared to CT. Breakdown or disruption of macroaggregates had resulted in decline of macroaggregates in Sc1 facilitating the decomposition of protected organic matter inside it. Our findings are in line with Gupta Choudhury et al. (2014) who also found positive effect of ZT on macroaggregation compared to CT. The macroaggregates are proved to be the rich conserver of organic carbon though highly prone to oxidation (Gupta Choudhury et al., 2014). Higher proportion of macroaggregates enhances carbon sequestration and nutrient availability which is manifested through good aeration and higher water infiltration within the root zone (Jat et al., 2018).

### 4.3 Relationships among the soil parameters and residue load under CA based managements

Higher residue load in different CA-based practices resulted higher WB-C, TOC through higher carbon input at upper soil layer and thereby improving the aggregate formation in soil. Interestingly, higher residue retention at upper soil layer facilitated the formation of macroaggregates rather microaggregates which is manifested in higher infiltration in soil in the same experiment (Jat et al., 2018). Similarly, formation of microaggregates as well as microaggregate associated C is lesser due to fewer disturbances in upper soil layer under full CA-based management practices. Bhattacharyya et al. (2013) observed higher macroaggregates as well as other aggregate indices such as MWD, GMWD, AR, AS under CA-based practices in Indian Himalayas. Gupta Choudhury et al. (2014) also observed higher macroaggregation and aggregate indices under similar production systems of Northwest India.

### 4.4 Variation in systems productivity

Higher system productivity (REY) in CA-based scenarios might be due to improved nutrient availability (Jat et al., 2018) through higher organic carbon enrichment in soil (Oldfield et al., 2019). Residue retention provides favourable micro-climatic conditions (Gathala et al., 2013), resulted in improved soil quality (Choudhary et al., 2018a, b). On individual crop wise, higher grain yield was recorded under transplanted rice than direct seeded rice. In direct seeded rice, proper crop establishment, weed menace, iron deficiency and unavailability of good cultivars are the possible reasons for lower yields (Kumar et al., 2018). But, on system basis, higher productivity was recorded with CA-based systems because of higher yield of succeeding wheat crop and integration of mungbean into the system. Pittelkow et al. (2015) in a global meta-analysis compared zero tillage with conventional tillage practices and found yield reduction in zero tillage, though in variable extent. But zero tillage when combined with residue retention and crop rotation, can produce equivalent or greater yields than conventional tillage, by minimizing its negative impacts. Moreover, zero tillage in combination with crop rotation and residue retention significantly increased rainfed crop productivity in dry semiarid/arid climates (Pittelkow et al., 2015). Lower system yield in Sc4 in 1^st^year than other years (Fig. 6), was probably because of late sowing of maize coupled with water logging and lower solar radiation in maize (Gathala et al., 2013).

## 5 Conclusion

This study showed the benefits of CA-based management practices on soil quality parameters especially in organic carbon in cereal based systems over passes of time. Higher soil organic carbon was recorded in CA-based management practices after six years of continuous cultivation. Significant improvement in soil aggregation, aggregate associated C and POC was observed with full CA-based management practices. Improved soil quality parameters facilitated in improving the systems productivity by ∼14% and 9% (on average basis) with CA-based maize-wheat-mungbean and rice-wheat-mungbean system over conventional rice-wheat system/ farmer’s practice, respectively. For long term soil and crop sustainability, CA-based management practices portfolios is an excellent option under cereal based systems of Northwest India. Therefore, CA based cultivation should be scaled out to whole IGP to check the soil quality deterioration through soil organic carbon build up while improving the crop productivity to achieve farmers’ posterity.
